# Scientific-intellectual movements in the post-truth age: The case of the Extended Evolutionary Synthesis

**DOI:** 10.1177/03063127251348254

**Published:** 2025-07-11

**Authors:** Sophie Juliane Veigl, Zinaida Vasilyeva, Ruth Müller

**Affiliations:** 1 Department of Philosophy, University of Vienna, Vienna, Austria; 2 African Center for Epistemology and Philosophy of Science, University of Johannesburg, Johannesburg, South Africa; 3 Department of Nature and Society, Museum für Naturkunde Berlin, Berlin, Germany; 4 Department of Science and Technology Studies, School of Social Science and Technology, Technical University of Munich, München, Germany; 5 Department of Economics and Policy, School of Management, Technical University of Munich, Munich, Germany

**Keywords:** scientific intellectual movements, post-truth, life sciences, evolution, Extended Evolutionary Synthesis

## Abstract

Over the past decade, evolutionary biology has seen an unusual number of heated debates centered around the pronouncement of an Extended Evolutionary Synthesis (EES). This theoretical framework brings together researchers from a range of disciplines in biology, such as ecology, developmental biology, and epigenetics, as well as philosophers of science, to challenge some of the key tenets of contemporary evolutionary theory, by arguing for a greater role of the environment and the organism in evolution. In this article, we analyze the EES as a scientific-intellectual movement (SIM) that has emerged under two specific conditions. First, evolutionary biology has always been both scientifically and socially influential and contested. As a field that claims to answer fundamental questions of how life has come to be, evolutionary biology has shaped causal thinking in fields as diverse as biology, psychology, and economics, and has influenced cultural thought and politics. Second, this specific contestation of mainstream evolutionary thinking emerges in the midst of challenges to particular sciences by what are seen as ‘post-truth’ and ‘anti-science’ movements. Drawing on interviews, participant observation, and document analysis, we examine the credibility strategies that EES proponents employ under these conditions, highlighting what happens when opponents of the EES make use of the ‘post-truth’ label to argue against the EES. We argue that this transposition of structures familiar from public and political debate onto contestations within science represents an important topic of study for STS researchers in the current political moment.

Exchanges between EES supporters and critics need not escalate into a mudslinging battle. Instead, researchers should be allowed simply to get on with their exciting science. Readers are recommended to make up their own minds about the legitimacy or otherwise of EES research. In this post-truth world, it becomes even more important that scientists stand up for rational inquiry, relying on evidence and their own reading of the literature rather than authoritative pronouncements. (Extended Evolutionary Synthesis, 2020)

Over the past decade, evolutionary biology has seen an unusual number of heated debates centered around the pronouncement of an Extended Evolutionary Synthesis (EES). The EES is a novel theoretical framework understood as ‘a way to think about and understand evolutionary phenomena that differs from the conception that has dominated evolutionary thinking since the 1930s (i.e., the modern synthesis)’ (Extended Evolutionary Synthesis, 2020). The EES brings together researchers from a range of fields within biology and beyond, such as ecology, developmental biology, epigenetics, and the philosophy of science, some of which fields have a long tradition of discontent with the current mainstream of evolutionary biology. Crucially, researchers from these fields have been criticizing what they see as the gene-centric nature of contemporary evolutionary biology, which views spontaneous genetic mutation and the natural selection of favorable traits that emerge from these mutations as the one dominant mechanism of evolution. The proponents of the EES, conversely, argue that many mechanisms, such as non-genetic inheritance or environmental induction, contribute to shaping the processes and outcomes of evolution in non-trivial ways.

One might assume that this attention to gene-environment interactions is a rather foreseeable development, given the current rise of the postgenomic life sciences, such as environmental epigenetics and RNA biology. In the past 30 years, postgenomic research approaches have promoted a new understanding of the genome not as self-contained and separate from the environment, but as deeply entangled with and responsive to it, across various fields in biology (Baedke & Fábregas-Tejeda, 2023; Richardson & Stevens, 2015). However, in evolutionary biology, there is considerable disagreement, and even frequent and fierce mudslinging, over this postgenomic challenge to the status quo. A letter opposing a Royal Society of London meeting on the EES has been said to state that holding this meeting was ‘similar to the Society allowing advocates of homeopathy to organize a meeting’.^1^ Opposing scientific articles linked the emergence of the EES, for example, to ‘increasing post-truth tendencies within science’ (Gupta et al., 2017), while more moderate skeptics argued that any reconsideration of the Modern Synthesis of evolution was unnecessary and that ‘all is well’ in evolutionary biology (K. Laland et al., 2014, p. 161). At the same time, proponents of the EES accuse researchers in mainstream evolutionary biology of being ‘hostile’, ‘emotive’, and ‘angry’ (Sridhar & Lala, 2024). and not responding to the EES scientifically but territorially. Thus, while postgenomic approaches to life seem to be establishing themselves in conversation with or at least peacefully alongside more traditional perspectives in most fields of biology, in evolutionary biology, the EES is causing significant controversy.

In this article, we explore the EES as a scientific-intellectual movement (SIM). Frickel and Gross (2005) coined this term to describe dissenting groups within science that, similar to social movements in society, seek to change the status quo in a field and to redistribute power, recognition, and resources. Frickel and Gross (2005) argue that

SIMs are collective efforts to pursue research programs or projects for thought in the face of resistance from others in the scientific or intellectual community. … the birth of a SIM often is marked by the announcement of a bold new intellectual program, and its death either by the effective disappearance of the movement from the intellectual scene or by its transformation into a more stable institutionalized form such as a school of thought, subfield, or discipline. (p. 208)

In their programmatic article, Frickel and Gross propose to study SIMs to, firstly, understand change in scientific fields and to, secondly, examine the processes by which dissent is handled in scientific communities. Frickel and Gross draw on numerous studies that detail the birth, life and death of such movements, some successful, such as T.H. Morgan’s drosophila genetics (Kohler, 1994) and some not, such as ‘psychologism’ in early 20th-century German philosophy (Kusch, 1995). To analyze these movements, they draw heavily on social movements theory, which they use to understand the social, epistemic, and political dynamics that emerge around SIMs. However, Frickel and Gross draw at least one clear distinction between social movements and SIMs that is crucial for our analysis. While they ‘take it as axiomatic that SIMs are influenced by direct or indirect pressures emanating from the broader cultural and political environment’ (p. 209), at the same time ‘SIMs are rarely contentious in the same way that political activism is’ (p. 226). While we agree that in most cases proponents of a SIM are, in general, much less likely to come under serious threat than political activists, we contend that this boundary is also blurrier than Frickel and Gross assume. Specifically, we show how a cultural climate of post-truth in which science finds itself regularly ‘under attack’ (Alexander, 2018) reshapes what it means to promote a SIM in science, and what is at stake for the individuals who do so.

## Evolution, science and society

Evolutionary biology occupies a unique position in its relations with other biological research fields, scientific fields beyond biology, and social and political discourse. It is frequently claimed that in biology ‘nothing makes sense except in the light of evolution’ (Dobzhansky, 1973). Evolutionary thought has heavily influenced reasoning in many scientific fields that explore the lives of organisms ranging from plant to animal and human, such as developmental biology (Laubichler & Maienschein, 2007), zoology (Mayr, 1999), physical and cultural anthropology (Fuentes, 2021), psychology (Griffiths, 2001), and the behavioral sciences (Longino, 2006), amongst others.

The importance of evolutionary biology for other fields and disciplines is often justified by distinguishing between proximate and ultimate causes (Mayr, 1961; Tinbergen, 1963): Whereas evolutionary biology can answer ultimate ‘why?’ questions, other fields and disciplines are often presented as catering to evolutionary biology by answering the proximate, often mechanistic ‘how?’ questions (Scott-Phillips et al., 2011; critical discussion in K. N. Laland et al., 2011). There is thus a history of what one might call a ‘disciplinary imperialism’ (Mäki, 2013) of evolutionary biology towards other fields and disciplines, assuming explanatory primacy for the ultimate reasons behind traits and behaviors.

Beyond its significance for biology and related scientific fields, historians of science have richly demonstrated that from its inception, evolutionary theory was tightly bound up with social and political theory (Bowler, 2013; Ruse & Richards, 2009). The intellectual history of Darwin’s *Origin of Species* foreshadowed and epitomizes these dynamics: Darwin wrote in his autobiography that his formulation of evolutionary theory was deeply inspired by Thomas Malthus’s *Principle of Population*, which proposes that the population will grow exponentially, while resources will not, resulting in an ever-increasing competition among individuals—an assumption that was soon falsified by the agrarian revolution and the steep increase in the production of food and other resources for human life.

However, it was not only political-economic theory that influenced evolutionary theory; evolutionary theory, in turn, significantly and repeatedly shaped 19th- and 20th-century social, economic, and political thought and policies. The most notorious example is social Darwinism (Radick, 2019), which transposes the Darwinian conceptualization of competition between individuals of the same species as the key formative force for biological development to the cultural and economic development of human society. Social Darwinists argued against socialism and the welfare state, and instead promoted social, economic, and political systems that forced individuals to compete with each other for income and security as this struggle would, ultimately, lead to innovation and the improvement of the human condition. This reading of evolutionary theory and its tight coupling with capitalist agendas ultimately resulted in the rejection of (neo-)Darwinism by the USSR, which in turn promoted a program broadly construed as Lamarckism and strongly identified with Soviet agronomist Trofim Lysenko (Meloni, 2016), which located the cause of evolutionary change in environmental adaptations, leading to experiments with plants, animals and humans in harsh climates that cost many lives (Lewontin & Levins, 1976).

Furthermore, evolutionary biology has been criticized frequently for its understandings of sex and gender, which have been argued to be heteronormative and based on Victorian gender stereotypes of active, conquering males and passive, coy females (e.g. Hubbard, 1979/2003; Monk et al., 2019). Narrow evolutionary perspectives on sex and gender still significantly influence the analysis of human and animal behaviors, promoting understandings of ‘normal’ and ‘abnormal’ behaviors for male and female animals (Roughgarden, 2013). Scientific accounts that normalize traditional human gender roles as biologically determined, in turn, are frequently cited by gender essentialists to argue against gender equality as well as queer and trans rights (Stryker & Chaudhry, 2022).

Evolutionary biology, then, has been a prime example of how biological explanations ‘do political work’ (Lock, 1999, p. 96). At the same time, evolutionary biology has also been rendered the focus of politically motivated attacks under the banner of creationism, particularly, but not exclusively, in the United States of America. Creationism rejects the notion that life could have evolved without the influence of a divine entity. Creationists particularly oppose the teaching of evolutionary theory in schools. They argue that evolutionary theory presents just one account of how life developed, on par with, e.g., religious narratives. In this sense, creationists are pioneers of contemporary anti-science and post-truth movements that work to render beliefs, feelings, and opinions on par with scientific theory and results. In her analysis of creationists’ arguments in the 1970s and 1980s, Nelkin (2000) concludes that creationists ‘frame their opposition to the teaching of evolution in ways that resonate with persistent public concerns about many aspects of contemporary science and fears about its pervasive influence on social and religious values’ (p. 536). Creationists thus turn science into a moral matter: What will it do to society and its moral fabric, if we believe that all life was created in random processes of mutation and selection? They thereby question over which realms of life science should hold authority (Toumey, 1991).

From the 1990s onwards, some creationists took a different route and began to use the label ‘intelligent design’ to argue against evolutionary theory. Using scientific vocabulary, proponents of intelligent design argue that evolutionary theory cannot completely account for how life has evolved and that only the actions of a divine force, an ‘intelligent creator’, can explain the timeframes and sophistication of evolution. Intelligent design is particularly important in the United States, where it is supported especially by conservative Evangelical Christians, who also hold significant influence in the Republican Party and have positioned the teaching of evolutionary theory in schools as a partisan issue alongside topics such as abortion legislation, gay marriage or trans rights. Proponents of intelligent design accuse evolutionary biologists of being unwilling to account for contradictions between data and theory in their field and suppressing well-founded critique (Nelkin, 2000, p. 538). Like contemporary anti-science movements promoting, for example, vaccine hesitancy, they cast mainstream science as dogmatic and untrustworthy. Today, both traditional creationism and intelligent design continue to expand their influence, particularly in the US, but also increasingly in European, Asian and African countries, referencing both Christian and Islamic beliefs to oppose evolutionary theory.

## Evolutionary theorizing in the 20th century

When critics—ranging from feminist STS scholars to creationists—engage with evolutionary biology, they usually address evolutionary theorizing based on the ‘Modern Synthesis’ (MS). First announced in 1942 (Huxley, 1942),^2^ the MS was a key part of efforts to recreate biology in the image of physics. Many researchers at this time believed that biology was lacking discipline and scientificity, because it did not have a unifying theory. The MS was thus imagined as a force to unify biological thinking through one overarching theory that would connect and reshape research across all its disparate subdisciplines (Smocovitis, 1992). In addition, it was to put a definite end to the so-called ‘eclipse of Darwinism’ (Bowler, 1983), a period roughly from 1880 to 1920 when core principles of Darwinism, such as natural selection, were under dispute and multitudes of competing theories emerged. With the formulation of the MS, evolutionary theorizing should finally be constrained to one theory that focused on random mutation and natural selection as its key mechanisms (Provine, 1988).

To create the MS, the ‘architects’ of the synthesis—amongst them evolutionary biologists Ernst Mayr and Theodosius Dobzhansky and paleontologist George Simpson—brought together the concept of Mendelian genetic inheritance with the Darwinian notion of natural selection. They further added a focus on population-level thinking to create an all-encompassing account of the evolution of all living organisms (Smocovitis, 1992). Historians of science argue that this also led to the mathematization of evolutionary theorizing and prioritized a particular set of empirical problems that could be solved through mathematical modelling (Cain, 2002). This effectively led to the emergence of a new research program with an agreed-upon set of problems, newly founded professional societies and journals, textbooks, as well as ‘rituals of celebration and commemoration’ (Smocovitis, 2020).

Since its inception, formulations of the MS have differed across time and epistemic contexts. However, two key tenets are always present: first, genetic mutation is the exclusive source of an unlimited and undirected variability of traits; and second, the natural selection of favorable traits is the only force that shapes the direction of evolution (Mayr, 1959). Evolutionary biology is often conceived as the science of understanding how these two forces interact and shape the direction of evolution.

In the second half of the 20th century, the discovery of the structure of DNA and the novel molecular methods for genetic analysis arguably strengthened a focus on genetics within the field. It is particularly this focus on genetics that has repeatedly garnered criticism throughout the second half of the 20th century. Critics have argued that it has increasingly led to the exclusion of disciplines such as developmental biology, zoology, ecology, and anthropology from the core of evolutionary biology (Lamm, 2011). Furthermore, critics, at times prominent researchers within the field, such as biologist Steven J. Gould (1983), have argued that a focus on genetics would blind evolutionary biology to evolutionary processes that are not primarily gene-driven. However, whether there have been such exclusions has been disputed by historians of science; for example, Smocovitis (2020) questions the narrative of the exclusion of evolutionary anthropology from the core of evolutionary biology.

Thus, the current, postgenomic wave of criticism in the form of the EES rekindles debates that are a century old. At the same time, the EES emerges at a quite specific social, cultural and political moment in time characterized by post-truth and anti-science sentiments. In what follows, we examine the EES as a SIM being established against both of these backgrounds, focusing particularly on the credibility strategies that its proponents are using to positions the EES as a legitimate theoretical program within evolutionary biology.

## Materials and methods

In this article, we analyze the EES as a scientific-intellectual movement in evolutionary biology. For our analysis, we draw on 14 interviews with EES researchers, participant observation including ethnographic interviews at conferences, and literature analyses of programmatic publications of the EES community as well as responses from opponents. Research for this article was conducted mainly in 2018 and 2019. In line with the SIM framework, our analytical focus rests on understanding the EES as a movement that seeks intellectual change and a redistribution of resources and reputation. Our analysis is thus not a classical controversy analysis that aims for symmetry between EES and mainstream evolutionary biology, but focuses primarily on understanding the dissenting movement and its practices.

In the course of our fieldwork, we observed, interreacted with and interviewed researchers who to varying degrees associate with the EES: All respondents supported the intellectual framework of the EES but differed in terms of their involvement in the community, ranging from key proponents to more loosely associated collaborators. Some interviewees were involved in the research project ‘Putting the Extended Evolutionary Synthesis to the test’, which plays a major role in both the proposition and the opposition to the EES as we explore it below, while others were not. Formal interviews were conducted with two women and twelve men, all of whom were tenured professors, reflecting the EES community’s general composition: It has a strong bias towards institutionally established, male, white researchers with overall high career security and scientific visibility. What varied significantly though was their fields of research, which included fields as diverse as computational biology, epigenetics, philosophy of science, and ecology. This also reflects the general composition of the EES community, which is made up of researchers from diverse fields within the life sciences, who work on various model organisms, and have been brought up in a variety of countries, institutions, and academic traditions. All of our respondents considered the postgenomic challenges to the dominance of genetics an important context for the EES. However, only some apply postgenomic approaches, e.g., epigenetics or RNA biology, in their own work, while others conduct mainly theoretical work or work focused on organismal biology.

Interviews were semi-structured and followed a peer-to-peer interview approach, a version of the active interview (Holstein & Gubrium, 2003) that is sensitive to the shared professional membership of both the interviewee and the interviewer in the academic community (Fochler et al., 2016; R. Müller & Kenney, 2014).^3^ Each interview lasted about 2-3 hours, which is a testament to the interviewees’ desire to discuss the EES and the controversies around it. The interviews consisted roughly of three parts. In the first part, we discussed how and why the researchers became involved with the EES. The second part focused on how they perceived the controversy around the EES and what they considered to be at stake scientifically in this controversy. In this part, we also discussed potential challenges, for example, to scientific careers, when opting to follow a SIM. Finally, in the third part, we asked our interviewees to discuss the role of evolutionary theory beyond science and the possible societal implications of the EES.

Beyond formal interviews, participant observations at conferences allowed us to also conduct more informal conversations as part of our fieldwork. This enabled us to also capture the perspectives of younger researchers interested in the EES community and their sense of what it might mean for their future work and careers to associate with this SIM. Conversations and other relevant observations were recorded as field notes in a field diary. Conference observations added to the interviews because conferences are important sites where scientific communities are formed, as ‘they offer the opportunity for achievements to be celebrated, reputations to be established, and … simultaneously reflect, and shape, the nature of a field’ (Stephens & Dimond, 2016, p. 313). Thus, conference observations allowed us to trace how the EES community presents and fashions itself as well as observe negotiations and disputes about its values, goals and limits.

We further analyzed a number of texts that reflect how the EES positions itself as a SIM within evolutionary biology and responses to it by opponents. We selected texts that were published in scientific journals aimed to present the EES and its merits to the scientific community. We further included texts in scientific journals by opponents who responded to the announcement of the EES and engaged in deconstructing proponents’ claims and credibility strategies. Beyond scientific articles, we included prominent webpages and blogs written by scientists that picked up on and engaged in the debate. We further included media articles in prominent media (such as *The Guardian* or *Quanta* magazine) that reported about the debate and in which proponents and opponents were invited to voice their arguments. Analysis of these texts allowed us to capture the public-facing aspects of the SIM and the opposition to it.

All materials were analyzed with a grounded theory-based approach, employing several rounds of open and focused coding as well as memo writing (Charmaz, 2006; Strauss & Corbin, 1997). Around 800 themes emerged inductively, which were organized into 50 overarching categories. Results from the analysis were regularly discussed in the research team to control for and improve intersubjective reliability. During analysis, a focus emerged on the EES as a scientific-intellectual movement that has been emerging in times of anti-science and post-truth sentiments. We attended to the movement’s ‘credibility strategies’ (Epstein, 1995), as they positioned their propositions vis-à-vis the dominant scientific regime, which is what all SIMs need to do, but also worked to distinguish themselves from anti-science movements that seek to undermine the legitimacy of evolutionary theory as such. The notion of ‘credibility strategies’ thus served as a sensitizing concept for the last steps of analysis. We thus analyzed what strategies the EES employs to legitimize their dissent with what they construct as the ‘mainstream’ of evolutionary biology and how these strategies are enabled, shaped and limited by the societal context in which this SIM has been emerging.

## The Extended Evolutionary Synthesis: Formation and key tenets

The EES emerged in the 2010s against the backdrop of both the longstanding political history of evolutionary theory and an uptick in anti-science rhetoric in the context of the 2016 US presidential election, the UK Pro-Brexit vote, as well as the rise of right-wing populist governments across the globe. Proponents of the EES argue that fields traditionally neglected by the MS have created enough data at odds with the MS that the latter needs revision. In particular, proponents of the EES have made a case for the importance of niche construction, developmental biases, non-DNA-based forms of inheritance, and cultural inheritance (e.g., Jablonka & Lamb, 2014; K. Laland et al., 2014; Pigliucci & Müller, 2010).

Despite their differences and origins across biological fields, these perspectives central to the EES challenge core tenets of the MS in similar ways. Firstly, they support a notion of evolutionary change that violates the core tenet that evolutionary change is driven exclusively by random mutations and the selection of beneficial traits that might result from these random mutations. For example, epigenetics researchers have proposed that organisms might respond to environmental changes with epigenetic alterations, which in turn change the three-dimensional DNA structure, rendering mutations in certain genes more likely than in others (Variale, 2014).

Second, and already implicitly inherent in the example above, these critical perspectives grant some agency to organisms in shaping their evolution. This agency might manifest itself on a biological level, for example, through an epigenetic response to environmental change that facilitates genetic changes, or on a behavioral level, when the actions of organisms modify their environment and that of their offspring in ways that create novel ‘niches’ for themselves with different evolutionary pressures and parameters for selection (Odling-Smee et al., 2013). Developing a new theory of evolution that brings in and accounts for these findings is the self-set core mission of the EES.

It is not a coincidence that this concerted effort to promote a critical revision of the MS took shape after the completion of the Human Genome Project. Especially the project’s limited results explaining health and illness in a straightforward manner led to a certain disillusionment with genetic research (Baetu, 2012; Fox Keller, 2010). Consequently and concurrently, research approaches such as epigenetics and RNA biology that had taken a backseat to genetics for a long time (and are highly compatible with EES type thinking), begun to gain credibility, influence and funding.

Concretely, the EES was formulated and announced in a series of meetings and publications. The first meeting took place in Altenberg, Austria in 2008 (Pennisi, 2008; Whitfield, 2008), organized by Massimo Pigliucci and Gerd B. Müller, who had before published on extending the synthesis (e.g., G. B. Müller, 2007; Pigliucci, 2007). Many of the EES’s central figures were present, such as niche construction theorist John Odling-Smee, evolutionary-developmental biologist Gerd B. Müller, and evolutionary biologist Günther Wagner. And notably, three philosophers of science attended the meeting—philosopher and biologist Massimo Pigliucci, philosopher of biology Alan C. Love, and epigeneticist and philosopher Eva Jablonka. All had published on the conceptual shortcomings of the MS.

An edited volume with contributions from attendees followed in 2010 (Pigliucci & Müller, 2010). This edited volume was published as a ‘companion’ volume to another publication, the fourth edition of Julian Huxley’s ‘Evolution—The Modern Synthesis’ (Huxley et al., 2010), edited by Pigliucci and G Müller. In their introduction (p. 7) to Huxley’s monograph, they state: ‘The idea of a new, Extended Evolutionary Synthesis that we advocate in the companion volume is as controversial today as the idea of the Modern Synthesis was in Huxley’s time.’ Thus, through the foreword as well as the juxtaposition of the two volumes, Pigliucci and Müller had positioned the EES as the rightful heir of Huxley’s evolutionary biology.

While the Altenberg meeting garnered some attention within the scientific community (Whitfield, 2008), the EES only really received wider attention when the journal *Nature* picked it up in 2014 as an emerging controversy and invited a commentary from proponents and opponents in the form of a ‘Yes and No’ opinion piece headlined with the question: ‘Does evolutionary theory need a rethink?’ (K. Laland et al., 2014). This *Nature* feature also prompted wider media attention to the EES. Subsequently, the Royal Society and the British Academy convened a joint meeting on the topic in 2016 (see Royal Society, 2016), despite significant backlash from some evolutionary biology researchers, showcasing a burgeoning interest in the EES in diverse communities in biology.

Around this time, a number of key proponents of the EES applied for and received a large grant from the US-based John Templeton Foundation (2019), with the title ‘Putting the Extended Evolutionary Synthesis to the test’. This grant of US $7.5 million significantly increased the visibility of the EES and allowed key proponents to conduct coordinated research on key tenets of the EES. The Templeton Foundation is a private philanthropic organization established in 1987 by Sir John Templeton, with the stated mission of exploring ‘big questions’ at the intersection of science, religion, and philosophy. It supports research in the life, natural, and social sciences, and the humanities, including theology, annually awarding about US $150 million in grant funding. While it has funded a broad range of scientific research, the Foundation has also attracted sustained criticism for blurring the boundaries between empirical science and religious belief. Its funding priorities often reflect a theological agenda, particularly in its promotion of research that supports the compatibility of science and religious worldviews. In the early 2000s, it became known that the Foundation had supported researchers who later became prominent proponents of Intelligent Design. It has also been listed as one of the largest funders of climate-change denialist research.^4^ The Foundation has since distanced itself from Intelligent Design, yet many researchers remain suspicious of the Templeton Foundation’s interest in bringing science and religion closer together. The EES community thus received significant criticism from other researchers in evolutionary biology, who accused them of taking funds from dubious sources to advance their cause. Nevertheless, the EES proponents later accepted a second, though much smaller, grant from the Templeton Foundation, entitled ‘Bringing the EES to the classroom’.

It is this situation in which we began our research on the EES: A community had formed, accrued resources to develop a research program, and had garnered some attention within the scientific and wider public. At the same time, the volume of criticism towards the EES was rising, too. Our analysis below traces the credibility strategies that the EES community developed to persist as a SIM in this specific situation. We first focus on how EES proponents have emphasized the continuity of EES-related thought throughout the 20th century, counteracting the notion of novelty or disruptiveness of EES thought. Second, we show how proponents position the EES as an important means to encourage debates in evolutionary biology and to keep them ‘scientific.’ Finally, we discuss the role of creationism and the EES’s affiliation with the Templeton Foundation. In particular, we discuss how EES proponents respond to claims that their movement was not scientifically valid and akin to post-truth tendencies in science. Here, we detail the limits of the EES’s credibility strategies and key challenges for this SIM emerging in the context post-truth and anti-science movements.

## Credibility strategies of the EES

### Reconstructing the past

Across the different sets of materials of this study, members of the EES community provided a remarkably homogenous narrative about the EES’s intellectual roots and its emergence. All emphasized a long tradition of thought of which, in their perspective, the EES was only the most recent instantiation. Contrary to what they perceived as mainstream framings that presented the MS as a longstanding scientific consensus in evolutionary biology, the proponents of the EES emphasized that EES-like thought was continuously present in evolutionary biology throughout the 19th and 20th century. Conversely, they framed the formulation of the MS as a temporary disruption of these intellectual traditions and debates within evolutionary biology, sparked by the post-WW2 desire to unify biology and reshape it in the image of physics. As one interviewee argued:

I guess what I’m trying to say is that this Extended Evolutionary Synthesis … has [a] very old tradition to it. And it’s the modern evolutionary synthesis that is a relatively new thing, especially … in the way that it’s sort of solidified. So, all you have to do is just go back to the olden writers in evolutionary theory, for example, Dobzhansky or even Ernst Mayr at the beginning, and just sort of read them, and then you will see the features of Extended Evolutionary Synthesis. (evolutionary biologist_number 1)

With statements like this, EES researchers oppose their critics’ narrative of the EES as an immature and attention-seeking project by researchers who want to draw undue attention to their own work. Instead, interviewees portray the EES as part of a continuous, century-old intellectual tradition and cast the formulation of the MS as a discontinuous, disruptive event. In addition to pointing out that EES-type thinking is older than the MS, they also argue that this kind of thinking is persistent throughout the 20th century despite the emergence of the MS. Thereby, they create a narrative about how the MS was never as established as its proponents present it to be, and that traditions of evolutionary thought were heterogeneous throughout the 20th century.

Furthermore, proponents of the EES emphasize that the ‘founding fathers’ of the MS, such as Huxley, Dobzhansky or Mayr, were not strictly opposed to some of the ideas which are nowadays associated with the EES. Rather, they propose that EES-type thinking became erased from the MS only later on as part of power struggles within evolutionary biology. This ‘hardening’ of the MS is often narrated in connection with the rise of genetics in the second half of the 20th century, a new and influential research field in biology to which, as they argue, evolutionary biologists increasingly hitched their wagons. As one interviewee explains:

And then in my view, it became a social issue. And it’s a power struggle about the right of interpretation, of influence, of authority, of funding, of all these issues, where the term the Modern Synthesis became more and more solidified. And I believe that you could say that, in the beginning, the Modern Synthesis wasn’t such a strictly genetic theory, but as it’s been called, it hardened over the time and ended up in a purely genetic enterprise. (evolutionary biologist_number 4)

As in many accounts in our interview data, this interviewee connects the hardening of the MS to the rise of molecular biology and specifically of genetics in the second half of the 20th century. Power and funding structures developed in ways that favored a molecularized, gene-centric version of evolutionary biology. Other approaches, which were rooted in developmental biology, ecology, or biological theory, became harder to fund. Similar processes of transformation towards the molecular have been described across various fields of biology and arguably had significant impacts on resource allocation (Morange, 2020). This is how one senior interviewee, working at the intersection of theoretical biology and the philosophy of biology, described the changes that happened in evolutionary biology during this time period:

And, so, what you needed was not [even] a unified front for your discipline but a unified front to argue with your administration that you shouldn’t take my office space away and give it to the new molecular biologists. (philosopher of science_number 1)

It is thus important to note again that the EES emerged in the aftermath of the human genome project. At that time, disillusionment with purely genetic approaches to understanding life emerged in science and, to some extent, more broadly in society. Other molecular but nongenetic approaches, such as epigenetics and RNA biology, began to garner attention, credibility, and funding. These approaches are much more compatible with EES thinking, as they emphasize the importance of studying interactions between organisms and environments in which genes play a role but do not dominate the interaction. In this sense, the EES was formulated at a moment in time when EES-type thinking was able to connect its agenda to a new set of molecular approaches that could compete with genetics both in terms of credibility and attractiveness for funding.

In this context, references to specific historical figures take on a particularly important role. One such figure is developmental biologist Conrad Hal Waddington, who is referenced as both the founder of epigenetics and a key figure in challenging the hardening of the MS. Waddington is known for his ‘epigenetic landscape’, a theoretical model highlighting the importance of developmental plasticity and responsiveness to environmental factors throughout the differentiation of a cell (Loison, 2019). Proponents of the EES often position Waddington as a forefather of the EES. They frame Waddington as a scientific genius, who, despite his early attention to developmental processes and epigenetics, was largely overlooked during his time. EES proponents often liken the resistance to their positions to the dismissal Waddington experienced. As one EES proponent put it:

My hero, Waddington, produced wonderful data for years, but it was explained away by, ‘Oh, that’s unimportant or a special case or, well, it’s a minor thing’, that kind of downplaying …. So, he had similar troubles all the way through. (evolutionary biologist_number 2)

EES proponents create a lineage between themselves and researchers whom they frame as intellectual ancestors and who are said to have challenged the mainstream biological thinking of their time. At the same time, by focusing on figures such as Waddington, who have been rediscovered in novel molecular fields such as epigenetics, they also hitch their wagon to the rise, rapid growth, and attractiveness of novel molecular research approaches that have begun to compete with genetics.

Frickel and Gross argue that it is pivotal for SIMs to create connections to historical figures and paint themselves as the heirs to their values, assumptions, and identities (Frickel & Gross, 2005, p. 223). The historical framing that EES proponents employ allows them to create a narrative of historical continuity and to draw boundaries towards mainstream evolutionary biology (see Petrović, 2022). The website of the key EES project ‘Putting the EES to the test’ also lists ‘The EES in historical focus’ as a sub-project of the research program (Extended Evolutionary Synthesis, 2020). Interestingly, there are currently no publications available from this sub-project; associated researchers include only one historian of science and three philosophers of science. Thus, there is an imbalance between historical narratives as a key part of the self-fashioning and credibility strategies of EES, and investments in scientifically understanding this history as part of the EES project.

In summary, in line with Frickel and Gross’s observations that historical narratives often play an important role for the construction of SIMs, we see that EES proponents employ a specific reading of the history of evolutionary biology as part of their credibility work. They narrate history in ways that: (1) point to a long and persistent intellectual history of EES-type thinking within evolutionary biology, including by the founders of the MS, (2) portray the MS as having ‘hardened’ over time through struggles for institutional power, gradually erasing this history of pluralistic thought, and (3) inscribe themselves into the history and present of novel, postgenomic molecular research approaches, such as epigenetics and RNA biology, that have begun to compete with genetics for funding, attention, and credibility.

### (Re-)defining scientificity, pluralizing science

The second aspect of the EES community’s credibility strategy strongly builds on their account of the heterogenous and contested history of evolutionary theorizing as well as their monolithic portrayal of the ‘hardened’ MS. EES researchers argue that the MS did not become dominant because it was uncontested, but because it, first, had, through its connection with genetics, easier access to resources, and, second, used this advantage to marginalize and discourage deviating thought and research. In short, EES researchers cast the mainstream of evolutionary biology as dogmatic and repressive of dissenting views. This is how one EES member recounts his experiences in the field:

There are several … people in the field who basically do nothing but police the neighborhood just to make sure there’s no dissenting views. (evolutionary biologist_number 4)

This quote is representative of statements by many interviewees who portray opponents as eager to eradicate any possible dissenting views. At the same time, interviewees frame the EES community as open and pluralistic. They underscore this point by showcasing that representatives of diverse biological disciplines participate in the EES and that the community furthermore would actively seek out researchers from non-biological disciplines, such as the philosophy of science, to take part in their debates. This is how one researcher framed the EES approach:

So as far as I am concerned, it is always healthy to have a pluralistic perspective, encourage people with different viewpoints. The rate at which the field progresses depends on the diversity of views and our capacity to turn those diverse views into alternative predictions and when we can test empirically and theoretically. (evolutionary biologist_number 3)

Drawing these two last quotes together, we see EES researchers employing three consecutive rhetoric moves here: first, they cast the proponents of the MS as dogmatic, and thus unscientific. Second, they frame the EES as a prototype for a modern research community that embraces pluralism and has heeded the call for innovation through interdisciplinarity. Thereby, the proponents of the EES develop an alternative narrative about epistemic authority: Rather than asserting mainly or merely tradition and consensus, they position pluralism, heterogeneity and debate as the key intellectual assets of the EES. Third, they cast this pluralism and debate with opposing views as essential aspects of scientific progress. Consequently, EES researchers do not understand why their claim that the MS needs a rethink has been met with such vehement opposition. One computational biologist associated with the EES argued, for example, that science progresses through assuming

a controversial point of view, let us say, an extreme point of view, and another person takes the opposite point of view, and you are willing to listen to each other’s arguments, you might at some point sort of learn from the other person certain aspects that leave you both in a better shape after the discussion. (evolutionary biologist_number 5)

Interviewees thus referred to a ‘Popperian’ ideal of science, highlighting the importance of conjectures and refutations for scientific progress. One interviewee pointed out with relish that Karl Popper, ‘the great logician of science, totally disagreed with the modern synthesis.’ (evolutionary biologist_number 2). This quote is not the only instance where EES-members bring into play meta-scientific positions. For instance, in their editorial note to Huxley’s MS, Pigliucci and Müller praise Huxley’s pluralist tendencies. This mobilization of positions about the sciences (such as scientific pluralism) pairs well with the involvement of philosophers of science, highlighting the contemporary and interdisciplinary character of the EES.

Proponents of the EES also use their emphasis on meta-scientific positions to criticize opponents. Many members of the EES community expressed surprise and disappointment that, in their perspective, the proponents of the MS did not hold up similar ideals and would not engage in a scholarly debate with them. They recounted how difficult it was to invite prominent proponents of the MS to the Royal Society Meeting in 2016 as well as to other meetings, and recounted the letter that a number of their opponents had written to the Society, likening the EES to homeopathy. One of the founding members of the EES argued that he considered proposing alternative scientific frameworks for understanding evolution as a perfectly normal scientific activity:

That is what scientists do … And I expected other scientists to react to what we had done in that way, just treating it as normal science. (evolutionary biologist_number 3)

Some EES proponents link what they frame as an extreme reaction to their propositions to what they perceive as the precarious status of theory in contemporary biology. In a few interviews, EES researchers argued that it was only through proposing the EES that they noted that some members of the evolutionary biology community had seemingly forgotten that the MS was a theory to which alternatives could be proposed. They mused that this might be an effect of the way biology was being taught in universities, mostly lacking courses in theoretical biology or the philosophy of science. This would result in students and researchers who lack the skills to critically assess the theory behind practices of data collection, evolutionary modeling, and the interpretation of results. This focus on theory is further underscored by the inclusion of philosophers of science in the core of the EES community; as well as by naming the first major EES grant, ‘Putting the Extended Evolutionary Synthesis to the test’, and focusing the grant narrative on testing theory against data. Proponents of the EES underscore the importance of theory for biological thinking, though they also insist that theory is not fact and criticize their opponents as having translated theory into dogma.

This focus on theory also shapes the practices by which key EES proponents police their own community members. Members who are perceived as ‘too extreme’ (evolutionary biologist_number 2), that is, who are not willing to engage scientifically with the content of the MS but rather reject it outright, have been facing harsh criticism, particularly if they act in ways that are deemed emotionally volatile or hostile at meetings or if they talk to the press in ways that the core members of the EES see as unscientific or disrespectful. Thereby, the EES membership actively limits what can count as an acceptable scientific persona: not the overly passionate, head-strong researcher who fights for their beliefs, but rather the ‘modest witness’ (Haraway, 1996) of early science lore, fashioned after an ideal of the British gentleman as humble and gentle, but also courageous and persistent. Keeping up such a demeanor, even when confronted with stark accusations or possible verbal abuse, becomes, at least in theory, one of the key features that is expected of a respected member of the EES community. The ideal proponent of the EES is a ‘polite revolutionary’: proposing alternative theories in line with recent findings, maintaining a pluralist attitude and not engaging in vile disputes with opponents.^5^

Yet, the figure of the polite revolutionary makes the status of the EES as a SIM a bit ambiguous. Building on Frickel and Gross (2005), Petrović (2022) differentiates ‘genuine’ from ‘stealth’ SIMs. Stealth SIMs ‘pursue change while emphasizing continuity … and cooperation’ (Frickel & Gross, 2005, p. 227) whereas genuine SIMs air high levels of grievances with what they perceive as the establishment (Petrović, 2022, p. 51). The EES, however, tries to navigate an in-between position’: On the one hand, EES proponents emphasize continuity, a mere ‘extension’ of what is already established and just ‘doing what scientists do’. On the other hand, the EES expresses high levels of grievances with establishment and seeks funding from an institution that is perceived as outside institutionalized science. In the next section, we explore how the context of ‘science under attack’ could partly explain this ambiguity.

In summary, the EES works to redefine scientificity in its credibility strategies through a number of rhetorical moves: (1) Proponents define scientificity as seeking out diverging opinions and creating pluralist, interdisciplinary communities to work together. (2) They contrast this perspective with their narrative about how they were met by mainstream evolutionary biology, whose rejection of invitations to discuss the EES they frame as mainly motivated by concerns about power and authority rather than about scientific progress. (3) They argue that they enact this understanding of scientificity by actively policing their own community, disciplining members who might act aggressively towards opponents and not uphold the image of the modest, open, and polite revolutionary that the EES seeks to promote.

### Reframing ‘science under attack’

In this last section, we discuss how proponents of the EES relate to and reframe the elephant in the room in any debate: creationism and, more recently, wider anti-science and post-truth sentiments. Critics usually refrain from openly likening the EES to creationism in written documents. However, on multiple occasions proponents of the MS have accused the EES of being unscientific in various ways. For example, in an article in *Quanta* magazine (Zimmer, 2016), Douglas Futuyma, a critic of the EES and the author of key textbooks in evolutionary biology, is quoted as saying that the appeal of the EES is largely emotional rather than scientific. He argues that the EES is appealing to some scientists and the public because it renders organisms an active force in their own evolution rather than just passive vehicles subjected to random mutations and selection. A scientific article about niche construction theory (Gupta et al., 2017), a key tenet of the EES, takes this criticism one step further. The authors argue:

We suggest that the manner in which niche construction theory is sought to be pushed in the literature looks more like an exercise in academic niche construction whereby, through incessant repetition of largely untenable claims, and the deployment of rhetorically appealing but logically dubious analogies, a receptive climate for a certain subdiscipline is sought to be manufactured within the scientific community. We see this as an unfortunate and increasing post-truth tendency within science. (Gupta et al., 2017, p. 493)

Both sets of claims show how proponents of the MS apply arguments usually reserved for conflicts at the interface between science and alternative knowledge cultures and belief systems to the EES community. Futuyama’s claim that the EES has a more emotional than scientific appeal is similar to claims about the premises and practices of alternative medicine or denials of climate change (Douglas & Sutton, 2015): They offer narratives that are more comfortable and comforting. The second stance goes one step further and explicitly accuses the EES of creating a post-truth discourse within science. Opponents thereby transpose discursive structures that readers would be familiar with from wider societal debates into the realm of science.

These kinds of arguments and insinuations are, unsurpingly, particularly prominent in online content and on social media. The most prominent online (as well as offline) critic of the EES is probably eminent evolutionary biologist and public intellectual Jerry Coyne, here summarizing his perspective on the EES on his personal website and blog ‘Why evolution is true’ with over 30,000 subscribers:

Well, you know, these ideas have been floating around for about fifteen years or more, and if the EES hasn’t proven itself productive, except in getting dosh to scientists and yielding an endless stream of speculative papers, maybe it’s time to reassess its value. But as long as Templeton keeps handing out millions of dollars to promote these ideas, there will be a never-ending stream of grant-hungry scientists with their hands out, eager to advance their careers by promoting the Templeton agenda. To my mind, this is the biggest example of misguided careerism I’ve seen in evolutionary biology over my lifetime.^6^

A leading figure in evolutionary biology, Coyne is also known for his public criticism of religion and especially of creationism. Here, he accuses the proponents of the EES of putting their career goals before scientificity and he characterizes the EES as promoting the ‘Templeton agenda’, which he argues elsewhere lies in ‘overturning the modern view of evolution’ (Coyne, 2009). While the Templeton foundation might no longer overtly support research tied to intelligent design, he argues that supporting EES research would serve the same agenda, which would ultimately lie in undermining science and blurring the boundaries between science and religion (see also Waldrop, 2011). In fact, this is also how the EES is interpreted by prominent Christian media, which assert that dissensus in evolutionary biology in the form the EES would show the fallacy of evolutionary thinking (e.g., Tomkins & Bergman, 2017).

Coyne’s blog also featured a highly positive review of Gupta et al.’s (2017) discussion of niche construction, though he distanced himself from their conclusion that the EES emphasis on niche construction would represent ‘post-truth tendencies’ within science. He framed this conclusion as ‘unnecessary and a bit mean’, though only after reprinting the passage in bold in his blog post. As we can see, Futuyama, Gupta et al., and Coyne have all made statements that frame the EES as outside of science and as blurring the boundaries between science and belief. The label post-truth circulates in these discussions and is either implicitly invoked—e.g., when Futuyama calls the appeal of the EES emotional rather than scientific—or is explicitly used as in the Gupta et al. article. In each case, the EES is framed as an epiphenomenon in evolutionary biology that does not merit engagement.

How do EES proponents respond to such criticisms? Some interviewees framed these responses as a ‘second hardening’ of the Modern Synthesis (evolutionary biologist_number 4), an updated form of maintaining power over what counts as relevant evolutionary biological research. Nevertheless, EES proponents felt that these accusations were making it difficult for their movement to be taken seriously, and particularly to become an attractive research field for younger researchers. Responding to these criticisms for many involved being active in the arenas where criticisms were launched most fervidly, i.e., different internet platforms. Founding members of the EES narrate that in order to ‘win the battle’ for credibility they had to ‘wise up’ (evolutionary biologist_number 3) and create webpages, hire science communicators, become more active on social media platforms and actively engage with journalists.

The project webpage of ‘Putting the EES to the test’ contains features that have developed in response to critics. For example, it features a dedicated section entitled ‘Why is the EES contentious?’ (Extended Evolutionary Synthesis, 2020). This section actively picks up various criticisms against the EES in a question-and-answer style manner, including post-truth accusations. Questions include ‘What motivates interest in the EES?’, ‘How can the EES seek “profound change” and yet disavow “revolution”?’ and ‘What is the contention surrounding John Templeton Foundation funding?’ This part of the homepage shows greater similarity to the webpages of social movements that seek to effect social change than to regular homepages of research consortia. Across the different answers to the featured questions, EES proponents mainly employ the credibility strategies that we have described, emphasizing historical continuity and the value of a pluralist approach to and open debate within science. In their answer to the final question of the section, ‘Why do EES critics, and advocates alike, complain that their opponents have an agenda?’, EES proponent explicitly seek to counteract the accusation of participating in a post-truth challenge to science. Instead, they frame the objectives of the EES as exactly the kind of scientific work that is needed in a post-truth world to restore confidence in science. As quoted at the very beginning of this article:

In this post-truth world, it becomes even more important that scientists stand up for rational enquiry, relying on evidence and their own reading of the literature rather than authoritative pronouncements.

While EES’s proponents do not turn the tables and accuse MS research of being post-truth, they do frame mainstream evolutionary biological research as dogmatic and authoritative, and for this reason untrustworthy, a common trope used by proponents of anti-science movements. We thus see that both sides use tropes common in post-truth and anti-science debates to undermine the credibility of the other party.

The credibility strategy developed by EES proponents in response to post-truth accusations shows a certain degree of ambiguity. While it involves insisting on the importance of open debate for scientific progress as well as for the public image of science, EES proponents, too, engage in tropes that are common in anti-science movements, i.e. arguments that the MS has hardened so much that it is no longer scientific. Furthermore, EES researchers accuse proponents of the MS themselves of having created targets for movements such as creationism and intelligent design by insisting that the MS could explain all of evolution and by ignoring diverging data. Furthermore, both sides have taken to using online media as key sites of debate.

Nevertheless, there are differences in the argumentation strategies of EES proponents and opponents. Accusations of dogmatism weigh differently than do accusations of post-truth practices. The scientific community is familiar with accusations of dogmatism. Yet, in the current cultural and political climate, accusations of engaging in post-truth practices carry with them associations with political forces that ultimately seek to undermine science as such. What does the availability of the rhetorical resource of accusations of ‘post-truth’ mean for the EES and possibly other SIMs? It is to this question to which we turn to conclude this article.

## Discussion and conclusion: SIMs in the post-truth age

In this article, we outlined the credibility strategies of the EES, a scientific-intellectual movement within evolutionary biology. We were interested in understanding how the EES as a SIM articulates itself and how the specific character of the field of evolutionary biology and the specific societal context of our time, characterized by concerns about anti-science and post-truth practices, shape these articulations.

First, proponents of the EES seek to render their movement credible by emphasizing a long and continuous history of EES-type thought while at the same time connecting their approach to novel developments in molecular biology, such as the rise of epigenetics and RNA biology. Second, EES researchers invoke the importance of scientific pluralism, open scientific debate and an understanding of theory, to propose that their approach is more scientific than that of the proponents of the MS. Each of these two strategies responds to specific criticisms: that the EES reflects the concerns of a small scientific minority who seek to draw undue attention to their work, and that their concerns and proposition are not worth debating because they are already covered well enough by contemporary evolutionary biology research.

Third, we discussed instances in which the EES has been accused of being akin to a post-truth movement within science. Here we see how the contemporary political context comes to matter for the crediblity work of this SIM. Because of intense public debates about the rise of anti-science and post-truth attitudes and movements in society, opponents of the EES can use the ‘post-truth’ label as a ready-made package of connotations and transpose them on to the debate about the EES. This ability to transfer multiple connotations at once sets apart the rhetoric strategy of likening the EES to ‘post-truth’ movement in science from usual practices of scientific criticism. As philosophers and historians of science have shown (e.g., Black, 1954; Stepan, 1986), analogies can transfer multiple associations. Through analogies ‘a “system of associated commonplaces” that strictly speaking belong only to one side of the metaphor are applied to the other’ (Stepan, 1986, p. 268).

By custom, criticism in science needs to engage with dissenting arguments one by one and invalidate them—a practice that is, for example, still visible in the *Nature* ‘Yes and No’ opinion piece ‘Does evolutionary theory need a rethink?’ (K. Laland et al., 2014). In contrast, the ‘post-truth’ label can attack the entire SIM at once and move it from within science to the outside, grouping it with various contemporary others of science, such as climate denialism, vaccine hesitancy, creationism, and general anti-science sentiment. By likening the EES to post-truth movements, opponents effectively create a shorthand that communicates that proponents of the EES—just like these other groups—are untrustworthy, politically motivated, deluded or unscrupulous careerists, and might hold questionable political views. At a moment in time when members of academia feel under attack, and the conduct of scientific work is threatened by hostile political climates and funding cuts, the post-truth analogy becomes a knock-down argument: an argument that silences debate before it has even begun, as a result of fears of being associated with that which is threatening science on a fundamental level.

What does it mean if discussions about what constitutes good or bad science venture into the terrain of accusations of post-truth stances? And what does it mean in a field as contentious as evolutionary biology? Historians of science have repeatedly shown that evolutionary theory, from its inception, has been both highly political and highly influenced by political and economic theory. In a day and age when social and natural science were not yet as divided as they are today, Darwin found no shame in explaining how Malthus’s socioeconomic account of the *Principle of Population* influenced how he translated his detailed observations in nature into theory. Yet, contemporary attempts to reformulate this theory, which has been highly influenced by the social, economic, and political contexts of its time and still shapes contemporary understandings of nature and society, are framed as politically motivated and consequently dismissed. This does not hold only for the EES. When Stanford professor Joan Roughgarden (2013) published a book that criticized the heteronormative nature of contemporary evolutionary biological theory, her work was accused of being politically motivated. As a trans-woman, she was accused of pushing her own agenda rather than developing a scientific critique. It stands to reason that the availability of the post-truth label will make it ever easier to dismiss such critique, and to group it with those others who are outside of science and whose criticism is not based in fact but in emotion, politics or delusion.

At the outset of this article, we quoted Frickel and Gross (2005) arguing that while they ‘take it as axiomatic that SIMs are influenced by direct or indirect pressures emanating from the broader cultural and political environment’ (p. 209), SIMs are different from social movements in that ‘SIMs are rarely contentious in the same way that political activism is’ (p. 226). While we do not want to suggest that political activism and promoting SIMs are the same, we are not certain that this distinction is as clear as Frickel and Gross make it out to be. On the basis of our analysis of the EES, we propose that the scientific and social roles of a research field can significantly influence how politically contentious a SIM can become and what is a stake for those who seek to advance it. Evolutionary biology, as we have outlined in the beginning of this article, has a history of fierce contestation about what rightfully constitutes the field and what constitutes political readings. Current contestations play out in front of anti-science and post-truth movements, with creationism and intelligent design aimed at evolutionary theory being elements of these movements. We thus propose that the analysis of SIMs requires a situated approach that considers how field specificities influence, and how changes in social context affect, the social and political life of a SIM.

In the specific context of the EES, societal context might explain the specific form the EES takes, particularly its ambiguous status. Its form might lie between a ‘genuine’, i.e., radically revolutionary and a ‘stealth’, i.e. cautiously transformative, SIM (Petrović, 2022). The looming presence of creationism, intelligence design and general anti-science sentiments affect how the EES can and cannot present itself as a movement opposing standard evolutionary theory. Asked about the EES in 2008 Jerry Coyne argued: ‘People shouldn’t suppress their differences to placate creationists, but to suggest that neo-Darwinism has reached some kind of crisis point plays into creationists’ hands’ (Whitfield, 2008). While Coyne makes space for criticism, he also makes clear that what constitutes ‘crisis rhetoric’ is left to be judged by eminent figures in the field—such as Coyne himself. This leads to fundamental questions about how much questioning is currently possible in evolutionary biology and possibly in similarly politically contentious fields, and how communities can respond differently to significant external pressures than by rigid forms of policing dissent. The availability of the post-truth label, and with it the easy transfer of multiple associations via analogy, should be considered a relevant factor in relation to these questions.

The emergence of post-truth sentiments has led STS scholars to debate how STS can study the construction of scientific facts in ways that are sensitive to this political climate (e.g., Collins et al., 2017; Sismondo, 2017a, 2017b). The EES presents a relevant case. More research should be done on how this and other SIMs articulate their dissent in a post-truth context. Interestingly, philosophers of biology associated with the EES (e.g. Gefaell & Saborido, 2022; Lewens, 2019) have not yet addressed the post-truth concern from within the EES.^7^ Furthermore, the case of the EES shows that counter-arguments to the post-truth label also draw on tropes that are common in (though not exclusive to) anti-science movements, such as accusing opponents of being dogmatic and authoritarian. We believe that it will be important for STS researchers to pay attention to how the rhetoric of post-truth enters scientific debates in the current moment and what social and epistemic dynamics it might entail. While we cannot, at this point, say anything about how the use of the post-truth label will influence the fate of this SIM or about how the controversy around the EES will settle, we suggest that following this and similar cases cases will be important for the study of how the post-truth context affects science in the long run.

## Notes

1. The letter was read to the authors during an interview.

2. See Provine (1980) for an exhaustive list of key publications between 1930 and 1954.

3. Peer-to-peer interviews acknowledge that interviewer and interviewee are both part of academia, a fact that needs to be reflected in the practice and analysis of the interviews. In particular, the interviewer aims to remain sensitive to how shared norms and values might influence the course of the interview, create specific silences or taboos or render certain practices and normative assumptions as given. The interviewer actively tries to probe these silences, inviting the interviewee into a shared process of reflection of latent values and established norms.

4. https://www.smithsonianmag.com/smart-news/meet-the-money-behind-the-climate-denial-movement-180948204/ retrieved 12.4.2025.

5. It is also this context in which proponents of the EES reject any insinuations that they aim to prove that Lamarck was right, because they perceive Lamarckianism as controversial, inherently political, and largely unscientific.

6. https://whyevolutionistrue.com/category/epigenetics/ retrieved 16.11.2024.

7. In this respect, it is interesting to note that, so far, the EES has largely not collaborated with philosophers who take a more ‘political’ approach to understanding science, such as feminist philosophers of biology or STS scholars who focus more on studying the social and political conditions of research than mainstream philosophy of science. The inclusion of philosophers of biology thus allows the EES to claim interdisciplinarity, while also being safe from more radical interventions, practices and thought processes from more political humanities.
